# The Three-Fold Axis of the HIV-1 Capsid Lattice Is the Species-Specific Binding Interface for TRIM5α

**DOI:** 10.1128/JVI.01541-17

**Published:** 2018-02-12

**Authors:** Damien Morger, Franziska Zosel, Martin Bühlmann, Sara Züger, Maximilian Mittelviefhaus, Benjamin Schuler, Jeremy Luban, Markus G. Grütter

**Affiliations:** a Department of Biochemistry, University of Zurich, Zurich, Switzerland; b Program in Molecular Medicine and Department of Biochemistry and Molecular Pharmacology, University of Massachusetts Medical School, Worcester, Massachusetts, USA; Ulm University Medical Center

**Keywords:** B30.2, CypA, HIV-1, TRIM5, TRIM5α, affinity, binding, capsid, interaction, species specificity

## Abstract

Rhesus TRIM5α (rhTRIM5α) potently restricts replication of human immunodeficiency virus type 1 (HIV-1). Restriction is mediated through direct binding of the C-terminal B30.2 domain of TRIM5α to the assembled HIV-1 capsid core. This host-pathogen interaction involves multiple capsid molecules within the hexagonal HIV-1 capsid lattice. However, the molecular details of this interaction and the precise site at which the B30.2 domain binds remain largely unknown. The human orthologue of TRIM5α (hsTRIM5α) fails to block infection by HIV-1 both *in vivo* and *in vitro*. This is thought to be due to differences in binding to the capsid lattice. To map the species-specific binding surface on the HIV-1 capsid lattice, we used microscale thermophoresis and dual-focus fluorescence correlation spectroscopy to measure binding affinity of rhesus and human TRIM5α B30.2 domains to a series of HIV-1 capsid variants that mimic distinct capsid arrangements at each of the symmetry axes of the HIV-1 capsid lattice. These surrogates include previously characterized capsid oligomers, as well as a novel chemically cross-linked capsid trimer that contains cysteine substitutions near the 3-fold axis of symmetry. The results demonstrate that TRIM5α binding involves multiple capsid molecules along the 2-fold and 3-fold interfaces between hexamers and indicate that the binding interface at the 3-fold axis contributes to the well-established differences in restriction potency between TRIM5α orthologues.

**IMPORTANCE** TRIM5α is a cellular protein that fends off infection by retroviruses through binding to the viruses' protein shell surrounding its genetic material. This shell is composed of several hundred capsid proteins arranged in a honeycomb-like hexagonal pattern that is conserved across retroviruses. By binding to the complex lattice formed by multiple capsid proteins, rather than to a single capsid monomer, TRIM5α restriction activity persists despite the high mutation rate in retroviruses such as HIV-1. In rhesus monkeys, but not in humans, TRIM5α confers resistance to HIV-1. By measuring the binding of human and rhesus TRIM5α to a series of engineered HIV-1 capsid mimics of distinct capsid lattice interfaces, we reveal the HIV-1 capsid surface critical for species-specific binding by TRIM5α.

## INTRODUCTION

The family of tripartite motif-containing (TRIM) proteins encompasses nearly 100 members, many of which play important roles in innate immunity and have antiviral activity. Of the approximately 20 TRIMs identified to inhibit retroviral infectivity, TRIM5α imposes the most potent block to retroviral infection (1). The discovery of TRIM5α as the main restriction factor responsible for the HIV-1 resistance in rhesus macaques (rhTRIM5α) has prompted intensive research efforts, making TRIM5 one of the best-studied TRIMs (2, 3). Many of the functional aspects of how TRIM5 acts to confer resistance to retroviruses can be attributed to its multidomain composition, characteristic of the tripartite/RBCC motif: an N-terminal RING domain, followed by a B-box2 domain and a coiled-coil domain (4). The RING domain confers E3 ubiquitin ligase activity (5), the B-box2 domain mediates self-association important for higher-order oligomerization (6–8), and the coiled-coil domain causes TRIM to form stable dimers (9–14).

At the C terminus, TRIM5α contains an additional B30.2 domain. This domain specifically recognizes retroviral capsids and defines the restriction specificity (15, 16). Recognition by TRIM5α occurs immediately upon exposure of the retrovirion core to the target cell cytoplasm at the early postentry stage of retroviral infection (2, 3). The interaction of the B30.2 domain with the capsid is weak but is potentiated through avidity effects due to dimerization and higher-order oligomerization of TRIM5α into a hexagonal lattice that is complementary to the hexagonal arrangement of retroviral capsids, ideally aligning arrays of B30.2 domains to interact with the repeating binding sites on the capsid surface (6–8, 17–19). By its specificity for the common hexagonal lattice pattern of retroviral capsids, TRIM5α can recognize a broad range of retroviral cores of highly variable primary structures (11, 20–27). This allows TRIM5α to remain restrictive despite the high mutation rate of retroviruses. However, the structural basis for capsid recognition at the crucial step of binding remains elusive.

In TRIM5α, the determinants for restriction specificity are located on four variable loops, v1 to v4, on the surface of the B30.2 domain. Single nucleotide polymorphisms within v1 to v4 can change the restriction specificities of TRIM5α between different primate populations or species. Unlike some nonhuman primate orthologues, human TRIM5α (hsTRIM5α) only weakly inhibits HIV-1 infection but potently restricts N-tropic murine leukemia virus (N-MLV) instead (21, 22). A single amino acid substitution from arginine to proline at position 332 of hsTRIM5α is sufficient to confer the ability to restrict HIV-1 (28). In some primate species, the B30.2 domain has been replaced by cyclophilin A (CypA), which binds to the HIV-1 capsid protein via an exposed proline-rich loop that overlaps with the predicted binding surface of the B30.2 domain (3, 29–33).

Restriction activity of TRIM5α can be blocked in cells by saturation with virus-like particles bearing properly assembled capsid lattices, but free capsid protein does not saturate restriction activity in any context (15). This is because significant binding of TRIM5α is detected only with assembled retroviral capsid lattices: TRIM5α has been shown to associate with capsid cores of virions that have their lipid membrane stripped off by detergents and *in vitro*-assembled capsids (8, 9, 15, 18, 19). In contrast, the interaction with soluble monomeric or dimeric capsid proteins is very weak: the dissociation constant (*K_d_*) of the rhesus TRIM5α B30.2 domain to a monomeric HIV-1 capsid protein has been estimated to be around 0.4 to 0.5 mM (29, 34). The specific sensing of a conserved lattice pattern is noteworthy, as TRIM5α tolerates not only large variations in capsid sequence but also large differences in the shape of the assembled capsid: TRIM5α binds both spherical (e.g., N-MLV) and conical (e.g., HIV-1) virions, as well as *in vitro* assemblies of capsid or capsid-nucleocapsid forming cylindrical tubes or even planar two-dimensional lattices (8, 9, 15, 18, 19, 22). The differences in surface curvature present in those capsid structures are accommodated by changes in the interhexamer spacing of the capsid lattice, while the hexameric and pentameric building blocks remain rigid (35). Therefore, it seems surprising that no dramatic increase in binding affinity was reported for isolated capsid hexamers relative to other capsid oligomers (34).

Although oligomerization through the B-box and coiled-coil domains greatly contributes to the binding affinity of TRIM5α, the monomeric B30.2 domain alone is sufficient to detectably bind to assembled capsid tubes (34). Atomic resolution structures of the TRIM5α B30.2 domain reveal that the predicted capsid-binding surface formed by the variable loops v1 to v4 is larger than the surface presented by one capsid subunit in the capsid lattice and suggest that the B30.2 domain binds to a surface area composed of multiple capsid subunits (34, 36).

In order to identify the binding site of rhTRIM5α on the HIV-1 capsid lattice, we measured the binding affinity of the TRIM5α B30.2 domain to several isolated capsid oligomers. Each oligomer mimics a specific high-symmetry capsid arrangement present in the hexagonal lattice, as we reasoned that B30.2 binding to these sites will allow the assembly of the complementary hexagonal TRIM5α lattice. We produced the full-length HIV-1 capsid protein, which forms dimers in solution, the monomeric capsid N-terminal domain (NTD), and a disulfide-stabilized capsid hexamer (37–39). To complete the collection of isolated HIV-1 capsid lattice mimics, we designed mutations that allow the production of a trimeric capsid surrogate that is cross-linked at the 3-fold symmetry axis of the lattice, reflecting the common interface between three adjacent hexamers. Furthermore, by comparing the binding affinities of the B30.2 domains of rhTRIM5α and hsTRIM5α to these HIV-1 capsid surrogates, we reveal distinct contributions of the mimicked capsid arrangements to the overall affinity and specificity of the restriction factor for the HIV-1 capsid lattice.

## RESULTS

### Design and production of a trimeric HIV-1 capsid that mimics the 3-fold interface on the HIV-1 capsid lattice.

**FIG 1:**
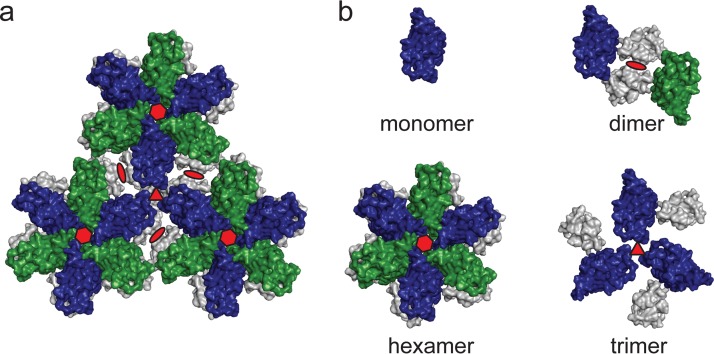
HIV-1 capsid lattice and oligomeric HIV-1 capsid surrogates that mimic distinct arrangements within the lattice. Models were generated by aligning the high-resolution structures of the HIV-1 capsid N-terminal domain (capsid NTD; PDB entry 1AK4) and the capsid C-terminal domain (capsid CTD; PDB entry 1A8O) into the low-resolution structure of two-dimensional capsid arrays (PDB entry 3DIK). (a) Excerpt of the hexagonal HIV-1 capsid lattice as represented by three neighboring hexamers. The 2-fold, 3-fold, and 6-fold symmetry axes of the lattice are indicated by red ellipses, triangles, and hexagons, respectively. The capsid NTDs are colored alternating in blue and green, and the capsid CTDs are shown in gray. (b) Models of oligomeric HIV-1 capsid surrogates used in this study to mimic distinct capsid arrangements at individual symmetry axes of the assembled HIV-1 capsid: the monomeric capsid NTD (monomer), the dimeric full-length wild-type capsid (dimer), the disulfide-stabilized hexameric capsid (hexamer), and the novel chemically cross-linked trimeric capsid (trimer).

In order to characterize binding of TRIM5α to the retroviral capsid surface, we produced oligomeric HIV-1 capsid molecules that mimic distinct capsid arrangements of the assembled HIV-1 capsid lattice (Fig. 1a): the wild-type, full-length HIV-1 capsid protein which forms dimers in solution (capsid dimer) (37), the monomeric HIV-1 capsid N-terminal domain (NTD) without the C-terminal dimerization domain (CTD) (capsid monomer), the disulfide-stabilized hexameric capsid (capsid hexamer) as described by Pornillos et al. (38), and a trimeric HIV-1 capsid that resembles an arrangement around the 3-fold axis of the capsid lattice (Fig. 1b). The interface at the 3-fold axis is formed by six capsid molecules: three of them form a trimeric contact via the CTDs, and the other three come into proximity at their NTDs. The proximal capsid NTDs form a surface that is accessible to TRIM5 binding, while the capsid CTDs are located on the inside of the HIV-1 capsid core.

**FIG 2:**
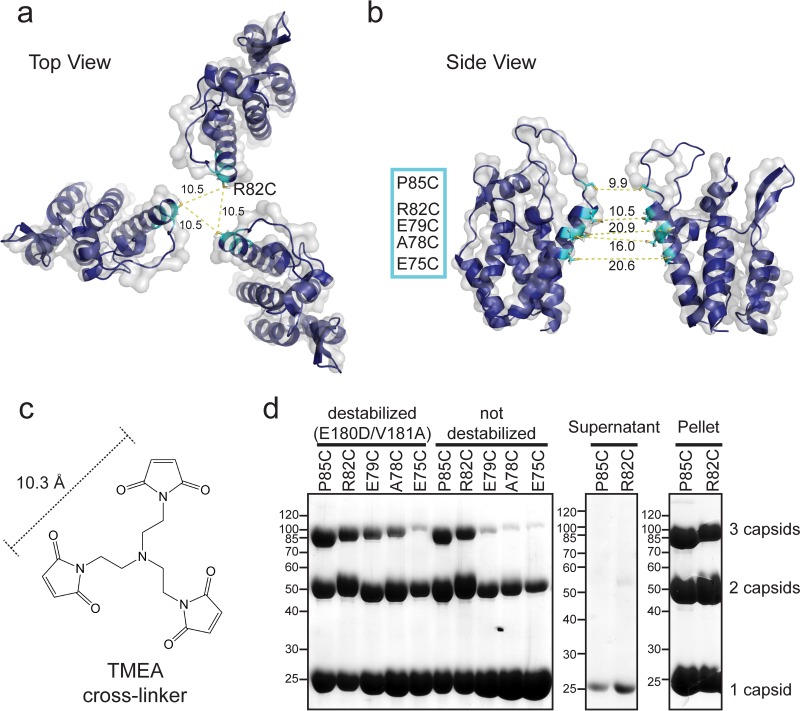
Cysteine mutations in the HIV-1 capsid NTD designed for cross-linking the 3-fold lattice interface. (a and b) Model of the trimeric lattice interface formed by the HIV-1 capsid NTDs, obtained by superimposing the high-resolution structure of the HIV-1 capsid NTD (cyan; PDB entry 1AK4) onto the cryo-EM-based model of planar HIV-1 capsid arrays (PDB entry 3DIK). Residues in close proximity between NTDs at the 3-fold axis were selected for mutagenesis to cysteines. Interdomain distances (in Å) between introduced cysteines (in cyan) in α-helix 4 and the CypA binding loop are indicated with yellow dashed lines. (a) Top view onto the 3-fold lattice interface formed by the capsid NTDs. (b) Side view of two NTDs in the 3-fold lattice interface. (c) The trifunctional maleimide TMEA with spacer arm distances of 10.3 Å used to cross-link the NTD-trimer interfaces via engineered cysteines. (d) SDS-PAGE analysis of *in vitro*-assembled HIV-1 capsid mutants chemically cross-linked with equimolar amounts of TMEA. Monomeric (1 capsid), dimeric (2 capsids), and trimeric (3 capsids) cross-linking products are indicated. The supernatant and pellet after centrifugation confirm that capsid trimers only form in the assembled capsid fraction, as shown for the mutants with the highest cross-linking efficiency (destabilized R82C and P85C mutants). Molecular mass standards in kilodaltons are indicated to the left of the Coomassie blue-stained SDS gels.

To design mutations that allow cross-linking of the capsid NTDs at the 3-fold lattice interface, available models (described below) of the assembled HIV-1 capsid shell were inspected for residues that come into proximity. These models revealed that residues within α-helix 4 have the closest distance between three adjacent capsid NTDs (Fig. 2a and b). However, according to the most recent model of the mature HIV-1 capsid (35) (PDB entry 3J34), these residues are located roughly 20 Å apart. This is too far for chemical cross-linking with commercially available trifunctional cross-linkers (Fig. 2c). However, this is not the case when the structure of a planar HIV-1 capsid lattice derived from two-dimensional capsid arrays is considered (40). There, the NTDs come closer together at the trimer interface due to the lack of curvature in the 2D lattice (PDB entry 3DIK). Also, TRIM5α is known to bind a broad range of retroviruses harboring different capsid curvatures, including planar hexagonal arrays formed by HIV-1 capsid-nucleocapsid mutants (18). Therefore, we considered the HIV-1 capsid lattice structure derived from planar two-dimensional (2D) capsid crystals a valuable model to design potential TRIM5-binding capsid oligomers. By aligning the crystal structure of the HIV-1 capsid NTD (PDB entry 1AK4) onto the cryo-electron microscopy (cryo-EM)-based 9-Å model of the planar lattice (PDB entry 3DIK) using PyMOL (33, 40), we estimated the closest distances to be ∼10 Å for residues R82 and P85 (Fig. 2a and b). This distance matches the requirements for cross-linking three neighboring capsid proteins with the trifunctional cross-linker TMEA [tris(2-maleimidoethyl)amine] (Fig. 2c). Residues E75, A78, and E79 along α-helix 4 were chosen as well for mutation to cysteines (Fig. 2b). Because TMEA cross-linking is performed on the assembled HIV-1 capsid, where all lattice contacts are formed, additional destabilizing mutations were introduced to facilitate subsequent disassembly of the cross-linked capsid lattice. Two sets of mutations, W184A M185A and E180D V181A, were tested to destabilize the dimerization interface in the capsid CTD (38, 41, 42).

**FIG 3:**
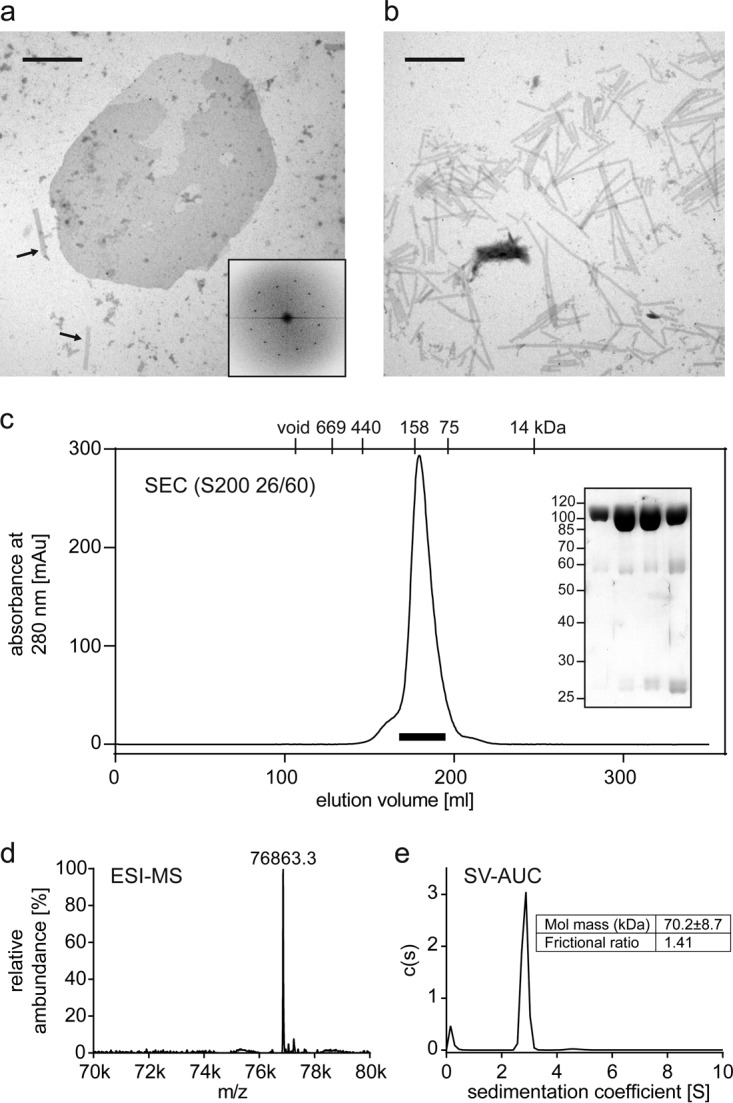
Production and biophysical characterization of the TMEA-cross-linked R82C E180D V181A capsid trimer. (a and b) Assemblies of HIV-1 capsid cysteine mutants that mimic the HIV-1 capsid surface and enable cross-linking of NTD-trimer interfaces. (a) Representative negative-staining EM image of *in vitro*-assembled R82C E180D V181A HIV-1 capsid. Assembly products form both elongated tubes, as seen on the bottom left (indicated by arrows), and planar sheets. One sheet is shown in the center of the micrograph. The scale bar corresponds to 1 μm. The hexagonal order of capsid molecules within an assembled HIV-1 capsid sheet is shown by a representative Fourier transform of a sheet image (inset). (b) Negative-staining EM image of *in vitro*-assembled R82C HIV-1 capsid lacking lattice-destabilizing mutations. This mutant forms elongated tubes only, as would be the case for the wild-type HIV-1 capsid (52). The scale bar corresponds to 1 μm. (c) Size exclusion chromatography (SEC) profile of the capsid trimer after disassembly of TMEA cross-linked R82C capsid lattices, with elution volumes of protein standards indicated on top of the panel. The SDS-PAGE analysis of 7-ml peak fractions indicated by the bar below the chromatography profile shows the high purity of the monodispersely eluting capsid trimer. The ESI-MS spectrum (d) and the sedimentation velocity analytical ultracentrifugation analysis (e) are consistent with the calculated mass of 76,866.3 Da for the HIV-1 capsid trimer. k, thousand.

To test the ability of these mutants to form the relevant lattice interfaces, recombinant capsid proteins were purified and tested for their ability to assemble *in vitro* by dialysis against 1 M sodium chloride at protein concentrations of 10 mg/ml. All capsid mutants, except for the ones harboring W184A M185A mutations in the CTD region, turned turbid, indicative of capsid assembly. Further analysis of the *in vitro*-assembled HIV-1 capsid variants by negative-staining EM revealed that cysteine mutants without destabilizing mutations formed elongated tubes, as observed for the wild-type capsid protein, while cysteine mutants combined with the destabilizing E180D V181A mutations formed both elongated tubes and planar sheets (Fig. 3a and b). The Fourier transform of negative-staining EM images confirms that these planar sheet structures are hexagonal arrays of assembled capsid molecules (Fig. 3a, inset). As outlined above, the planar geometry brings the introduced cysteines closer together, which increases cross-linking efficiency. Short incubation of the assembled capsid with equimolar concentrations of TMEA and subsequent disassembly yielded capsid oligomers coupled to only one TMEA molecule. Under these conditions, the R82C and P85C mutants with predicted distances of 10 Å cross-linked more efficiently to trimers than the other mutants with more distantly positioned cysteines (Fig. 2d). Separation of assembled capsids and soluble capsid proteins was done by centrifugation. Cross-linked trimers were found only in the pellet fraction, confirming that cross-linking of trimer interfaces occurs only in the hexagonal capsid lattice (Fig. 2d).

Of the R82C and P85C mutants, which cross-linked more efficiently to trimers, we focused on the R82C mutation for further purification and characterization, because modifying P85 in the exposed cyclophilin A binding loop might impair TRIM5α binding (33). Thorough biophysical characterization of the final TMEA cross-linked product (R82C E180D V181D) purified from cross-linked and then disassembled capsid confirmed that it is a trimer in solution (Fig. 3).

### Binding affinity of TRIM5α to the capsid dimer does not correlate with restriction specificity.

**FIG 4:**
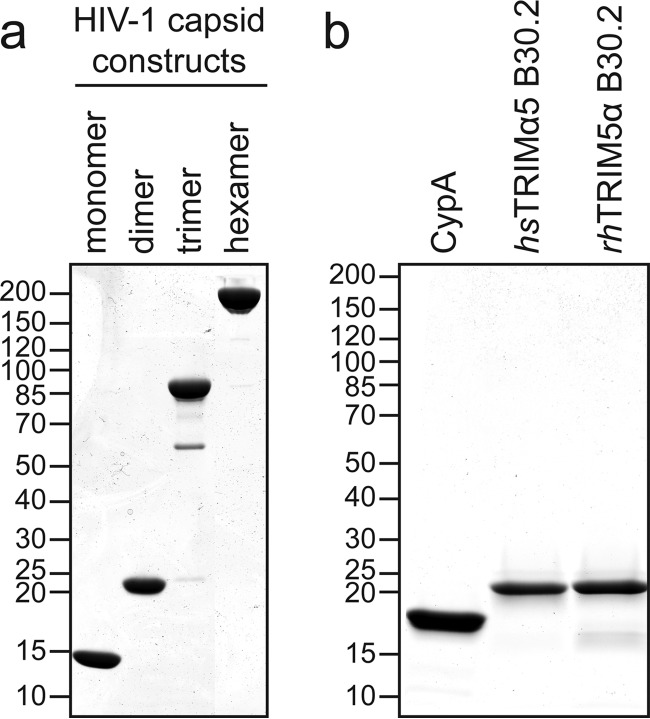
SDS-PAGE analysis of purified HIV-1 capsid molecules and fluorescently labeled capsid binding domains. (a) Nonreducing SDS-PAGE of purified HIV-1 capsid molecules. The capsid monomer (capsid NTD), the capsid dimer (wild-type, full-length capsid protein), the capsid trimer (full-length capsid proteins cross-linked via the NTDs), and the capsid hexamer (disulfide-linked full-length capsid proteins) show the expected relative increases in molecular mass. Note that the capsid dimer is dissociated into its two full-length subunits. (b) Reducing SDS-PAGE of purified and Alexa 488-labeled capsid-binding domains: the Flag-tagged CypA, the B30.2 domain of hsTRIM5α, and the B30.2 domain of rhTRIM5α. (a and b) Molecular mass standards in kilodaltons are indicated to the left of the Coomassie blue-stained SDS gels.

To measure binding affinities, we used microscale thermophoresis (MST) and dual-focus fluorescence correlation spectroscopy (2fFCS). The B30.2 domains from TRIM5α and its TRIM20 homolog were fluorescently labeled and titrated with increasing concentrations of unlabeled capsid molecules (see Fig. 4 for SDS analysis of the purified constructs). For determination of affinities in the micromolar range, fluorescence-based methods have the advantage that one reaction partner can be kept constant at a very low (nanomolar) concentration. Thus, the titration can be described with the simple binding isotherm equation *f_b_* = [capsid]/*K_d_* + [capsid], facilitating the comparison of results (*f_b_* is the fraction of bound B30.2 domain, [capsid] is the capsid concentration, and *K_d_* is the dissociation constant). All *K_d_*s reported in this work refer to the concentration of monomeric capsid equivalents to facilitate comparison between the individual values.

**FIG 5:**
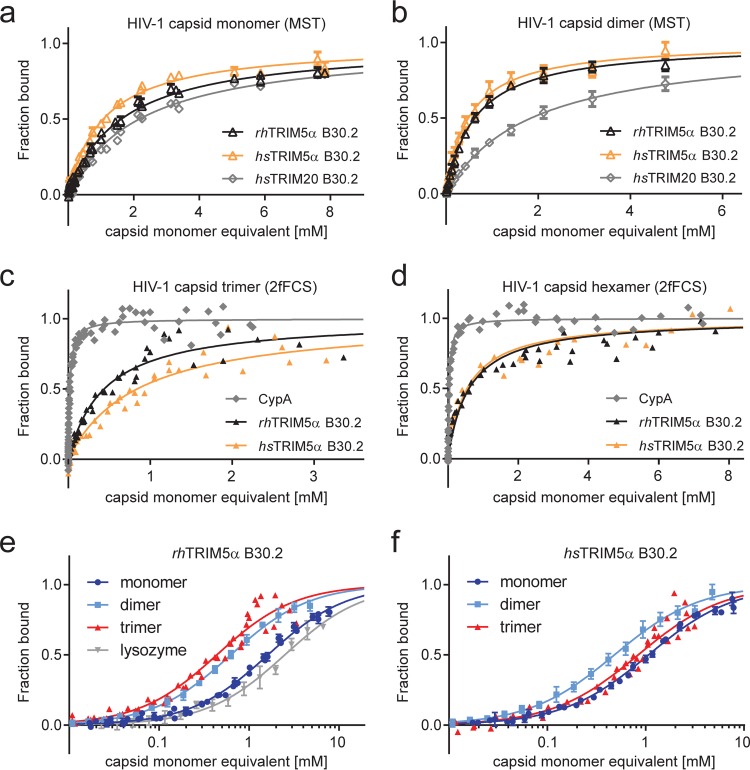
Binding measurements of TRIM B30.2 domains interacting with surrogates of the HIV-1 capsid lattice. The capsid concentrations are plotted as concentrations of monomeric capsid equivalents. All binding curves were fitted with a 1:1 binding isotherm to obtain the dissociation constant, *K_d_* (Table 1). (a and b) Comparison of the binding curves obtained from MST measurements of rhTRIM5α, hsTRIM5α, and hsTRIM20 B30.2 domains titrated with the capsid monomer (a) and dimer (b). (c and d) Measurements of capsid trimer (c) and hexamer (d) binding to the B30.2 domains of rhTRIM5α and hsTRIM5α by 2fFCS. For determination of the dissociation constants (*K_d_*), CypA was used as a reference to correct for the viscosity increase at high capsid surrogate concentrations (see Materials and Methods). (e and f) Comparison of binding curves obtained from MST and 2fFCS measurements for the B30.2 domains of rhTRIM5α (e) and hsTRIM5α (f). The titration of lysozyme to the B30.2 domain of rhTRIM5α is shown in gray and is representative of an unspecific interaction. The titration curve for the capsid hexamer is omitted for clarity.

By MST, migration of a fluorescently labeled protein is measured along a temperature gradient as a function of a titrated unlabeled binding partner to determine binding affinities. This method is not affected by the increased viscosity at high capsid protein concentrations, as it measures interactions via changes in the equilibrium distribution of the concentration (43). We performed MST measurements with the wild-type HIV-1 capsid dimer and homologous TRIM B30.2 domains. The rhTRIM5α B30.2 domain has the strongest affinity for the wild-type HIV-1 capsid dimer, in comparison to other capsid molecules reported so far (29). The measurements revealed a *K_d_* of 0.62 ± 0.04 mM for binding of the rhTRIM5α B30.2 domain to the capsid dimer. The B30.2 domain of TRIM20/pyrin, which has no restriction activity against retroviruses (1), was used as a negative control and exhibited a significantly weaker *K_d_* of 1.8 ± 0.1 mM for the capsid dimer (Fig. 5b).

**FIG 6:**
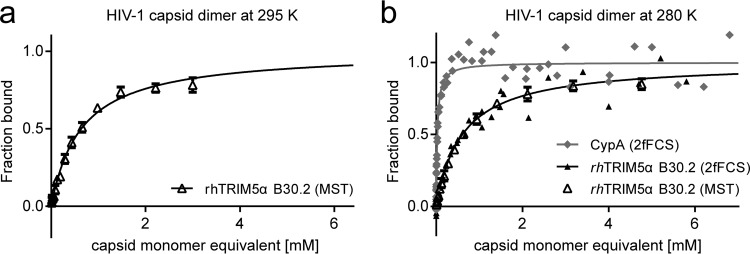
rhTRIM5α B30.2 binding to the capsid dimer measured at different temperatures and with different methods. (a) MST measurements performed at 295 K. Error bars indicate the standard deviation of three independent measurements. Fitting the data with equation 1 (black line) yields a *K_d_* of 0.64 ± 0.06 mM, within error of the *K_d_* obtained at 280 K. (a) Overlay of 2fFCS and MST measurements at 295 K. The MST data are fitted with equation 1 (solid line); error bars indicate the standard deviation of three independent measurements. For determining the *K_d_* by 2fFCS, CypA was used as a reference to correct for viscosity effects at increased capsid protein concentrations (see Materials and Methods). The 2fFCS data were converted to fraction bound with equation 3 and are shown with a 1:1 binding isotherm (solid line), calculated using the *K_d_* from a fit with equation 2. MST and 2fFCS yield consistent binding curves with *K_d_* values of 0.62 ± 0.04 mM (MST) and 0.60 ± 0.07 mM (2fFCS).

All measurements were conducted at 280 K because of aggregation of highly concentrated capsid surrogate solutions at higher temperatures. We ruled out a large influence of temperature on the binding affinity by comparing measurements at 280 and 295 K, which yielded very similar *K_d_*s (0.64 ± 0.06 mM at 295 K) for binding of the rhTRIM5α B30.2 domain to the capsid dimer (Fig. 6a).

To see if the *K_d_*s measured by MST correlate with the species-specific differences in HIV-1 restriction activity, we also measured binding of the capsid dimer to the B30.2 domain of hsTRIM5α. Unexpectedly, hsTRIM5α B30.2 had a slightly higher affinity for the capsid dimer (*K_d_* = 0.49 ± 0.06 mM) than its primate orthologue (Fig. 5b). It thus appears that the binding affinities toward the capsid dimer do not correlate with the *in vivo* HIV-1 restriction specificities of the two TRIM5α orthologues, suggesting that species specificity is determined by B30.2 binding to other capsid surfaces.

To determine whether capsid dimerization via the CTDs contributes to the affinity for the B30.2 domain, we performed MST measurements with the monomeric capsid NTD (capsid monomer). A very weak *K_d_* of 1.6 ± 0.1 mM was determined for the rhTRIM5α B30.2 domain, which is similar to the *K_d_* of 2.2 ± 0.1 mM obtained for the TRIM20 negative control (Fig. 5a). In addition, when lysozyme was used as a control for unspecific binding to rhTRIM5α B30.2, fitting of the apparent binding curve resulted in a *K_d_* of 2.6 ± 0.3 mM, which is in a similar range as that for the TRIM20 control (Fig. 5e; Table 1). Compared to the binding affinity of 0.62 mM per binding site for the capsid dimer (Fig. 5e; Table 1), the almost 3-fold lower affinity for the capsid monomer confirms the contribution of capsid dimerization to rhTRIM5α B30.2 binding previously reported by nuclear magnetic resonance (NMR) (29). Again, MST measurements of the capsid monomer interaction with hsTRIM5α B30.2 revealed a slightly higher affinity (*K_d_* of 1.1 ± 0.1 mM) than that for the rhesus orthologue (Fig. 5a and f).

**TABLE 1:** Overview of dissociation constants measured by MST and 2fFCS for the interaction of TRIM B30.2 domains and CypA with the different HIV-1 capsid surrogates normalized to monomeric capsid equivalents

Capsid surrogate or negative control	*K_d_*^*a*^			
	rhTRIM5α	hsTRIM5α	hsTRIM20	CypA
Capsid monomer	1.6 ± 0.1 mM^*b*^	1.1 ± 0.1 mM^*b*^	2.2 ± 0.1 mM^*b*^	ND
Capsid dimer	0.61 ± 0.04 mM^*d*^	0.49 ± 0.06 mM^*b*^	1.8 ± 0.1 mM^*b*^	26.5 ± 5 μM^*c*^
Capsid trimer	**0.44 ± 0.04 mM**^*c*^	**0.84 ± 0.09 mM**^*c*^	ND	17.8 ± 2 μM^*c*^
Capsid hexamer	0.60 ± 0.09 mM^*c*^	0.54 ± 0.1 mM^*c*^	ND	28.9 ± 3 μM^*c*^
Lysozyme	2.6 ± 0.3 mM^*c*^	ND	ND	ND

a The *K_d_*s for the interactions of TRIM B30.2 domains with the different HIV-1 capsid surrogates, as well as lysozyme, are indicated relative to the concentrations of the monomeric capsid equivalents. The *K_d_*s of the rhTRIM5α and hsTRIM5α B30.2 domains for the capsid trimer, which has the strongest binding affinity for the B30.2 domain of rhTRIM5α as well as the greatest increase in affinity compared to the *K_d_* for the B30.2 domain of *hs*TRIM5α, are highlighted in bold. ND, not determined.

b *K_d_* determined by MST.

c *K_d_* determined by 2fFCS.

d Average of *K_d_*s determined by MST and 2fFCS.

**FIG 7:**
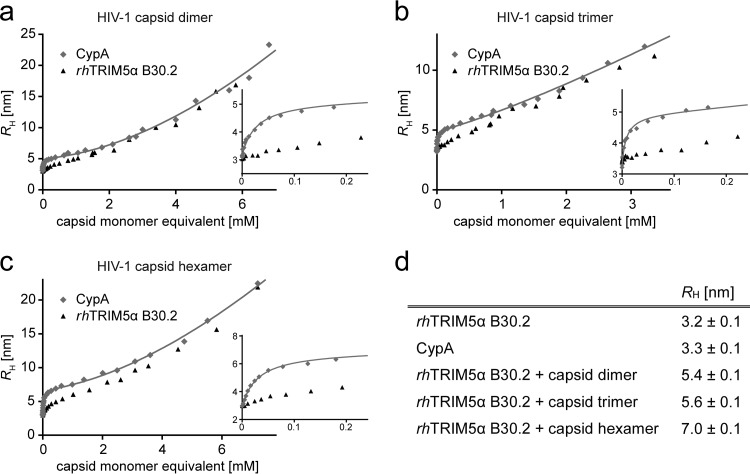
The hydrodynamic radius, *R_h_*, of rhTRIM5α B30.2 and CypA as a function of the concentration of HIV-1 capsid surrogates. *R_h_*, as determined by 2fFCS (see Materials and Methods), is plotted against the concentration of capsid dimer (a), trimer (b), and hexamer (c). Insets show concentrations of up to 0.2 mM capsid monomer equivalents to highlight the difference between B30.2 and CypA binding. The data are fitted with equation 2 (solid lines). For CypA, the two processes contributing to an increase of *R_h_* (capsid binding and viscosity increase) occur over well-separated capsid concentration ranges, allowing the reliable determination of *R_h_* of the CypA-capsid complex and the capsid concentration-dependent increase in viscosity (see Materials and Methods). (d) *R_h_* values determined for rhTRIM5α B30.2, CypA, and the rhTRIM5α/CypA-capsid complexes. Error bars indicate the standard deviation of three independent measurements. The *R_h_* values of CypA and rhTRIM5α B30.2 are identical within error, justifying the assumptions described in Materials and Methods. The hydrodynamic radii of the rhTRIM5α B30.2-capsid surrogate complexes are assumed to be identical to those of the respective CypA-capsid surrogate complexes.

To verify these rather low determined affinities and because other capsid surrogates than the capsid monomer and dimer could not be measured by MST, we performed independent measurements of the interaction between the B30.2 domain of rhTRIM5α and the capsid dimer by 2fFCS. With 2fFCS, binding affinities are determined by measuring the diffusion time of a fluorescently labeled protein whose hydrodynamic radius (*R_h_*) increases upon binding as a function of the concentration of the unlabeled binding partner (44). Since increasing solution viscosity at high capsid protein concentrations contributes to the binding-dependent change in diffusion time, human cyclophilin A (CypA) was measured as a reference in an independent experiment to correct for viscosity effects as described in Materials and Methods. CypA binds to the capsid surrogates with a much higher affinity (*K_d_* = 16 μM) (45) than rhTRIM5α, allowing us to separate the contributions of binding and viscosity to the change in *R_h_* (Fig. 7a).

In the absence of capsid protein, we measured an *R_h_* of 3.2 ± 0.1 nm for the rhTRIM5α B30.2 domain, which increases to 5.4 ± 0.1 nm (Fig. 7d) when the capsid dimer is bound. The *K_d_* of this interaction is 0.60 ± 0.07 mM, which matches the *K_d_* obtained by MST (Fig. 6b; Table 1).

### Species specificity of TRIM5α binding to the capsid trimer.

We next probed the interaction of the HIV-1 capsid trimer and hexamer with the TRIM5α B30.2 domains. The capsid hexamer recapitulates capsid interfaces at the 6-fold axis of the hexagonal lattice, and the capsid trimer reflects the common interface between three adjacent hexamers (Fig. 1). Since both capsid oligomers could not be measured to high enough concentrations by MST due to aggregation caused by the temperature gradient, we used 2fFCS as described for the capsid dimer to determine the binding affinities. We obtained *R_h_* values of 5.6 nm and 7 nm for the capsid trimer- and the capsid hexamer-B30.2 complexes, respectively (Fig. 7b to d).

The *K_d_* of the capsid hexamer for the B30.2 domain of rhTRIM5α was 0.60 ± 0.09 mM (Fig. 5d), showing that the capsid hexamer has an increased affinity for the rhTRIM5α B30.2 domain compared to the monomeric capsid NTD, but not compared to the capsid dimer (Table 1). To investigate if binding to the capsid hexamer accounts for the difference in HIV-1 restriction activity between hsTRIM5α and rhTRIM5α, we measured the interaction with the B30.2 domain of hsTRIM5α. However, these measurements revealed an affinity of 0.54 ± 0.1 mM, which is within error identical to the *K_d_* found for rhTRIM5α B30.2 (Fig. 5d and f; Table 1). We note that the data for the capsid hexamer did not perfectly fit the simple one-to-one binding model, as was the case for the other capsid surrogates (Fig. 5d). Possible reasons include the presence of an additional binding mode for the B30.2 domain or further oligomerization of the hexamer at higher concentrations.

Binding of the B30.2 domain of rhTRIM5α to the capsid trimer had a *K_d_* of 0.44 ± 0.04 mM (Fig. 5c). Thus, the capsid trimer shows the strongest increase in affinity relative to the monomeric capsid NTD among the capsid surrogates investigated (Table 1; Fig. 5e). In contrast, measurement of the interaction with hsTRIM5α B30.2 revealed a significantly lower affinity, with a *K_d_* of 0.84 ± 0.09 mM for the capsid trimer (Fig. 5c and e; Table 1). The affinity difference of the B30.2 domains of hsTRIM5α and rhTRIM5α for the capsid trimer correlates with the species-specific difference in restriction activity against HIV-1 and suggests that determinants for species-specific recognition by TRIM5α are located at the 3-fold lattice interface of the HIV-1 capsid.

## DISCUSSION

The capsid molecules described in this study mimic different surface areas on the assembled HIV-1 capsid core. All together, they represent all high-symmetry capsid arrangements on the hexagonal lattice. To determine the potential binding site of TRIM5α, we measured the binding affinities between these capsid surrogates and the B30.2 domains of rhTRIM5α and hsTRIM5α. A detailed knowledge of this binary interaction is essential for understanding the overall binding capability of TRIM5α to the HIV-1 capsid lattice, but the experimental determination of the very high dissociation constants poses a technical challenge. We measured these low affinities with two fluorescence-based methods, MST and 2fFCS. Thus, we could employ low concentrations of one titrant (the fluorescently labeled B30.2 domains), enabling the use of a first-order binding model to fit the data.

MST measurements revealed a *K_d_* of around 0.6 mM for binding of the rhTRIM5α B30.2 domain to the capsid dimer, which was confirmed by 2fFCS (Fig. 5a). This interaction is clearly distinguishable from the negative controls and the *K_d_* for the capsid monomer (Fig. 5; Table 1). This observation agrees with previous findings from NMR studies: the interaction with the capsid monomer is very weak, and dimerization of the capsid via the CTD greatly enhances the affinity for the B30.2 domain of rhTRIM5α (29, 34). We also found that relative to the capsid monomer, the affinity of the capsid dimer and hexamer for the B30.2 domain of rhTRIM5α is similarly increased (*K_d_* values of 0.62 and 0.60 mM, respectively) (Table 1). Strikingly, the capsid trimer, which has never been investigated before, had the highest affinity for the B30.2 domain of rhTRIM5α, with a *K_d_* of about 0.44 mM per capsid subunit (Fig. 5c and e; Table 1).

In order to investigate if one of these HIV-1 capsid surrogates mimics a lattice surface area that accounts for species-specific restriction by TRIM5α, we also measured the binding affinities of the hsTRIM5α B30.2 domain. We found that the capsid monomer, dimer, and hexamer all had a similar or slightly stronger affinity for the human orthologue (Fig. 5; Table 1). However, the capsid trimer had a significantly weaker affinity for the hsTRIM5α B30.2 domain than its rhesus orthologue (*K_d_*s of 0.44 mM and 0.84 mM, respectively) (Fig. 5c; Table 1). Based on this *K_d_* difference, we conclude that the capsid trimer harbors the binding surface area that contributes to the species-specific restriction potency of TRIM5α against HIV-1. The 2-fold increase in affinity between monomeric B30.2 domains for the 3-fold capsid lattice interface is expected to be potentiated through avidity effects in the context of HIV-1 restriction *in vivo*: the weak B30.2-capsid interactions are amplified, as TRIM5α self-assembles into a hexagonal superlattice matching the geometry of the assembled HIV-1 capsid, thereby ideally positioning many B30.2 domains to their repeating binding sites on the HIV-1 capsid lattice (6–9, 11, 18, 19, 36, 46). Therefore, small differences in affinity between different B30.2 domains will be translated into large differences in the overall binding capability of TRIM5α, which could define the restriction potency of the different protein variants. It is possible that the R82C mutation in α-helix 4 of the capsid trimer impairs B30.2 binding by influencing the capsid surface region near the CypA binding loop, explaining the weak affinities determined with the capsid trimer. Nevertheless, CypA binding to the capsid trimer was not weakened compared to the binding to the wild-type capsid dimer (Table 1), and we found that rhesus TRIM5α B30.2 binding to the capsid trimer was not just retained but was even stronger than the affinities measured with the other HIV-1 capsid surrogates.

**FIG 8:**
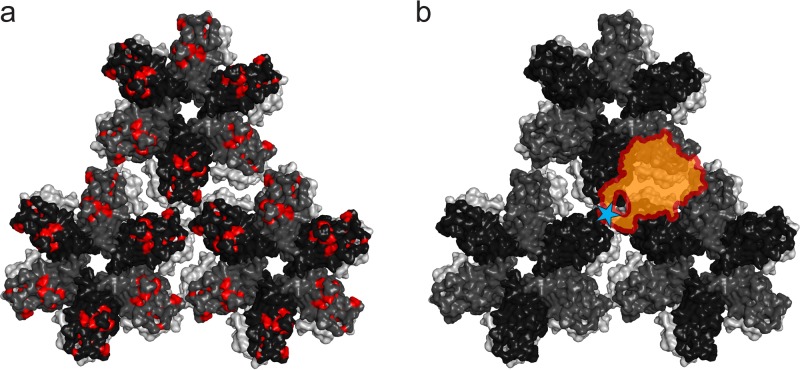
Model of the B30.2–HIV-1 capsid interaction. (a) Mapping of mutations on the HIV-1 capsid lattice that allow the virus to escape restriction by rhTRIM5α. These hot spot residues, predicted to be involved in the interaction with TRIM5α, are shown in red. (b) Model of the B30.2–HIV-1 capsid interaction that matches the binding data and involves the hot spot residues. The position corresponding to proline 332 in rhTRIM5α, which determines the species specificity of restriction compared to hsTRIM5α, is indicated by a blue star on the v1 loop of the B30.2 domain and is placed such that the v1 loop binds to the trimer interface of the capsid lattice. The rest of the B30.2 domain of rhTRIM5α, involving loops v2 to v4, is located such that it binds to the interface between two neighboring capsid hexamers. This interface combines the surface areas mimicked by the capsid dimer, the capsid trimer, and the capsid hexamer, matching the increased affinities measured for all oligomeric capsid arrangements compared to that of the capsid monomer.

Our data suggest that TRIM5α binds to an overlapping surface area shared among the capsid dimer, trimer, and hexamer, involving multiple capsid molecules. Such capsid arrangements are found at the interhexamer interfaces of the HIV-1 capsid lattice. Binding of TRIM5α to this region would be in line with the mapping of residues involved in TRIM5α binding to the outer edges of the capsid hexamer (26, 29–32) (Fig. 8a). On the B30.2 domain, capsid binding is predicted to be mediated by four variable loop regions, v1 to v4 (29, 47, 48). Recent atomic resolution structures of the TRIM5α B30.2 domain have shown that these loop regions form a continuous surface, large enough to cover three or four capsid molecules (34, 36). Hot spots of positive selection through primate evolution and residues most affected by capsid binding in NMR studies cluster in the v1 loop of the B30.2 domain of rhTRIM5α (47, 49). Also, the single arginine-to-proline mutation that switches the restriction activity of hsTRIM5α toward HIV-1 is located on the v1 loop (28). Based on our binding results, we propose a model for the interaction of the B30.2 domain of rhTRIM5α with the HIV-1 capsid lattice that involves these known binding determinants of TRIM5α. The B30.2 v1 loop and the capsid trimer interface are the dominating determinants for both affinity and specificity in the TRIM5α capsid interaction. Therefore, we suggest that the B30.2 v1 loop region binds near the 3-fold symmetry axis between three adjacent capsid hexamers of the HIV-1 capsid lattice. By positioning the remaining predicted binding surface of the B30.2 domain, involving the v2, v3, and v4 loops, at the 2-fold symmetry axis between two neighboring hexamers, we match the increased affinity observed for all the oligomeric capsids tested, as this interface combines capsid arrangements mimicked by the capsid dimer, trimer, and hexamer (Fig. 8b). A very similar binding model has been predicted by computational docking of the B30.2 domain to the HIV-1 capsid (50).

In the TRIM5 variant TRIMCyp, which confers HIV-1 resistance, for example, in owl monkeys, the C-terminal B30.2 domain has been replaced by a CypA domain (3). A binding model where the CypA domain preferentially binds at the 3-fold symmetry interface of the HIV-1 capsid lattice is also suggested by our data: as for the B30.2 domain of rhTRIM5α, we found that CypA has a higher average binding affinity per capsid subunit for the capsid trimer than for the other capsid molecules (Table 1). This is likely the case because the CypA binding loops on the HIV-1 capsids come together at the 3-fold symmetry axis, while in the dimer and hexamer they are pointing away from each other. Preferential binding of the CypA domain to the 3-fold capsid symmetry interfaces, as we propose for the B30.2 domain of TRIM5α, would allow TRIMCyp to match the same geometrical criteria to bind and form a complementary superlattice on the retroviral capsid as TRIM5α.

In conclusion, we present a novel chemically cross-linked HIV-1 capsid trimer that recapitulates the capsid arrangement at the 3-fold symmetry axis on the outward-facing side of the assembled HIV-1 capsid lattice. Its potential as a tool for characterizing HIV-1 capsid binding molecules is exemplified in this study. The binding measurements with orthologous TRIM5α B30.2 domains reveal that this capsid trimer has a stronger affinity for rhTRIM5α than other capsid oligomers that mimic capsid arrangements on the HIV-1 lattice. Of the monomeric, dimeric, trimeric, and hexameric capsids measured, only the binding affinities of the capsid trimer to the human and rhesus B30.2 domains correlate with their respective HIV-1 restriction activities. Based on our results, we propose a binding model that explains how a single point mutation in the v1 loop of the B30.2 domain is sufficient to confer HIV-1 restriction activity to the otherwise unrestrictive hsTRIM5α.

## MATERIALS AND METHODS

### Design and production of cross-linked HIV-1 capsid trimers.

The DNA sequence encoding amino acids 133 to 363 of the HIV-1 Gag polyprotein was amplified from psPAX2 (51) and cloned into a pET-20b vector (Invitrogen). Mutations were introduced by QuikChange mutagenesis (Invitrogen) and verified by DNA sequencing (Microsynth). The HIV-1 capsid proteins were expressed and purified as previously described (45), with the addition of 100 mM β-mercaptoethanol (βME) to all buffers. Capsid tubes were assembled *in vitro* by overnight dialysis at 4°C (52) into assembly buffer (20 mM Tris-HCl, pH 8, 1 M NaCl, 100 mM βME).

For cross-linking of assembled capsid tubes harboring mutations E75C, A78C, E79C, R82C, or P85C in their NTD combined with or without destabilizing E180D V181A mutations in the CTD, the initial assembly buffer was exchanged against cross-linking buffer [20 mM HEPES-HCl, pH 7.2, 1 M NaCl, 0.5 mM Tris(2-carboxyethyl)phosphine (TCEP)] by a two-step dialysis. Chemical cross-linking of neighboring NTDs via the introduced cysteines was performed at a concentration of 10 mg/ml capsid protein by incubation with the trifunctional maleimide cross-linker TMEA (Thermo Scientific) at a 1:1 molar ratio of TMEA to capsid monomer for 5 min on ice, followed by quenching with 10 mM dithiothreitol (DTT). Cross-linking products were analyzed by reducing SDS-PAGE.

For purification, TMEA-cross-linked capsid assemblies were disassembled by dialysis into disassembly buffer (20 mM Tris-HCl, pH 8) at 4°C, and the cross-linking products were separated by size exclusion chromatography on a Hi-Load Superdex 200 26/60 column equilibrated with disassembly buffer. Reinjection of the trimer-containing fractions onto the size exclusion column yielded essentially pure capsid trimers. Protein concentration was determined by UV absorbance using molar extinction coefficients at 280 nm (ε_280_) of 33,524 M^−1^cm^−1^. Extinction coefficients in this work are given for the protein monomers.

### Production of the HIV-1 capsid monomer, dimer, and hexamer.

For the production of monomeric HIV-1 capsid, the gene sequence encoding amino acids 133 to 278 of the HIV-1 Gag polyprotein, comprising the NTD of HIV-1 capsid, was cloned into a pET20b vector with a C-terminal His_6_ tag, cleavable by 3C protease. His-tagged HIV-1 capsid NTD was expressed as previously described (53) and purified by immobilized-metal affinity chromatography (IMAC) using Protino Ni-nitrilotriacetic acid (Ni-NTA) agarose (Macherey-Nagel). Briefly, E. coli cells expressing His-tagged HIV-1 capsid NTD suspended in lysis buffer (50 mM Tris-HCl, pH 8, 300 mM NaCl, 10 mM imidazole, 1 mM phenylmethylsulfonyl fluoride [PMSF], 10 μg/ml RNase A, 5 μg/ml DNase I) were lysed using an EmulsiFlex C3 homogenizer (Avestin, Canada). The cleared lysate was loaded on an equilibrated Ni-NTA gravity column and washed with 50 mM Tris-HCl (pH 8)–300 mM NaCl–40 mM imidazole, and bound protein was eluted with 50 mM Tris-HCl–50 mM NaCl–300 mM imidazole. The C-terminal His tag was cleaved by the addition of His-tagged 3C protease and dialysis against 50 mM Tris-HCl (pH 8)–50 mM NaCl–10 mM imidazole. Untagged HIV-1 capsid NTD was then separated from the tag and the protease by another IMAC and further purified by size exclusion chromatography on a Hi-Load Superdex 75 26/60 column equilibrated with 20 mM Tris-HCl, pH 8.

The wild-type HIV-1 capsid dimer and the disulfide-linked A14C E45C W184A M185A HIV-1 capsid hexamer were produced according to published procedures (38, 45).

Protein concentrations were determined using ε_280_ values of 24,980 M^−1^cm^−1^, 33′524 M^−1^cm^−1^ and 28′210 M^−1^cm^−1^ for the HIV-1 capsid NTD monomer, the HIV-1 capsid dimer, and the disulfide-linked HIV-1 capsid hexamer, respectively.

### TEM analysis of capsid assemblies.

Aliquots from capsid assemblies were diluted to 0.25 to 0.5 mg/ml, and 6-μl samples were incubated on Formvar-coated copper grids for 30 s. The grids were blotted, negatively stained three times on 20-μl drops of 2% uranyl acetate for 10 s, blotted, and air dried. Samples were imaged on a Philips CM 100 transmission electron microscope (TEM) at a magnification of ×13,000.

### ESI-MS of cross-linked capsid trimers.

Nanoelectrospray ionization mass spectrometry (nanoESI-MS) analysis of the samples was performed on a Q-TOF Ultima API mass spectrometer (Micromass, United Kingdom). The solutions were infused through a fused silica capillary (inside diameter [i.d.], 75 μm) at a flow rate of 0.50 μl min^−1^. Electrospray Pico Tips (i.d., 30 μm) were obtained from New Objective (Woburn, MA). Mass spectra were acquired by scanning an *m/z* range from 500 to 2,500 with a scan duration of 1 s and an interscan delay of 0.1 s. Spray voltage was set to 2.1 kV, cone voltage to 35 V, RF lens 1 energy to 50 V, and collision to 10 eV. Before injection, the samples were desalted using C_4_-ZipTips (Millipore) from which they were eluted with 10 μl of 50:50:0.01 (vol/vol/vol) CH_3_OH:H_2_O:HCOH (pH ∼2).

### Sedimentation velocity analytical ultracentrifugation.

The oligomeric state of cross-linked capsid proteins after purification was verified by sedimentation velocity analytical ultracentrifugation (SV-AUC) in 20 mM Tris-HCl, pH 8, at a protein concentration of 0.5 mg/ml. Sedimentation velocity measurements were conducted at 30,000 rpm and 4°C using a ProteomeLab XL-1 ultracentrifuge (Beckman Coulter). Data were analyzed with SEDFIT (54).

### Production of recombinant TRIM B30.2 domains.

DNA coding for the B30.2 domains of hsTRIM5α and rhTRIM5α (residues 286 to 493 and 288 to 497, respectively) was cloned into the pFXGST vector using fragment exchange (FX) cloning (55). The pFXGST vector was generated by modification of the MultiBac vector pFBDM (56) multiple cloning site 1 (MCS1) to enable FX cloning in analogy to that described by Weinert et al. (12). Briefly, the SapI restriction site between the transposon element Tn*7*R and the Col-E1 origin of replication was removed, and an NdeI restriction site was inserted into the MCS1 by QuikChange mutagenesis (Invitrogen). The FX cassette of the pBXGST vector (55) was used as the template (including the coding region for an N-terminal His_10_ tag, followed by glutathione *S*-transferase [GST] and a 3C cleavage site) and cloned into the modified pFBDM vector using NdeI and XbaI restriction sites. The NdeI restriction site was later removed by QuikChange, yielding the pFXGST vector.

The TRIM B30.2 domains were expressed in Sf21 cells using the manufacturer's protocol (Invitrogen). Cells were harvested after 65 to 72 h of expression at 27°C and lysed in lysis buffer (50 mM Tris-HCl, pH 8, 300 mM NaCl, 10 mM imidazole, DNase I, RNase I, Benzonase, and EDTA-free complete inhibitor cocktail [Roche Diagnostics]) using an EmulsiFlex homogenizer. Lysate was cleared, and the HisGST-tagged TRIM B30.2 domain in the supernatant was purified by gravity-flow IMAC as described above, followed by overnight dialysis (Spectra/Por; 12,000 to 14,000 molecular weight cutoff [MWCO]) in cleavage buffer (50 mM Tris-HCl, pH 8.5, 150 mM NaCl, 0.2 mM TCEP) at 4°C in the presence of a 1:20 (wt/wt) ratio of 3C protease. Cleaved HisGST tag and His-3C protease were removed by reapplying the sample to Ni-NTA agarose. The flowthrough was collected and concentrated to 2 mg/ml, as determined by UV absorbance using a ε_280_ value of 38,390 M^−1^cm^−1^ or 39,880 M^−1^cm^−1^ for the hsTRIM5α or rhTRIM5α B30.2 domain, respectively.

The B30.2 domain of TRIM20 was prepared as described by Weinert et al. (57) and concentrated to 2 mg/ml (ε_280_ = 32,430 M^−1^cm^−1^).

### Production of recombinant human cyclophilin A.

The human cyclophilin A (CypA) gene was cloned into the p7XC3H vector, which encodes a C-terminal 3C cleavage site and a His_10_ tag, using FX cloning. The PCR primers were designed such that a Flag tag is encoded at the N terminus of the CypA gene.

The CypA construct was expressed in E. coli Bl21(DE3). Cells were grown in 2× YT medium at 37°C to an optical density at 600 nm (OD_600_) of 0.8, and expression was induced with 0.5 mM IPTG (isopropyl-β-d-thiogalactopyranoside). Cells were harvested after 4 h of expression and lysed in 50 mM Tris-HCl (pH 8)–100 mM NaCl–0.5 mM TCEP–10 mM imidazole–1 mM PMSF–10 μg/ml RNase A–5 μg/ml DNase I using an EmulsiFlex homogenizer. Lysate was cleared, and the supernatant was applied onto a gravity-flow IMAC column as described above. The His tag was cleaved overnight at 4°C by dialysis (Spectra/Por; 6,000 to 8,000 MWCO) in the presence of 3C protease in 50 mM Tris-HCl, pH 8–100 mM NaCl–0.5 mM TCEP–10 mM imidazole and removed by reapplying the sample to the IMAC column. As a final purification step, the sample was applied to a Superdex 200 10/300 column equilibrated with 50 mM Tris-HCl (pH 8)–100 mM NaCl–0.5 mM TCEP–10% glycerol. Fractions containing Flag-tagged CypA were pooled, concentrated to 2 mg/ml using an Amicon Ultra-15 concentrator (3,000 MWCO; Millipore), and stored at −80°C. Protein concentrations were determined using a ε_280_ of 9,970 M^−1^cm^−1^.

### Fluorescent labeling of TRIM B30.2 domains and CypA.

Purified B30.2 domains and CypA were dialyzed (Spectra/Por; 6,000 to 8,000 MWCO) overnight at 4°C in labeling buffer (25 mM sodium carbonate, pH 8.5, 100 mM NaCl, 0.5 mM TCEP). Labeling was carried out on ice in the presence of a 1.2-fold molar ratio of NT-647-NHS (Nanotemper) or Alexa Fluor 488-NHS (Alexa 488; Thermo Scientific) by overnight incubation in the dark. At pH 8.5, labeling of the N-terminal amine (pK_a_ ∼8) is favored over the reaction with ε-amines of lysines (pK_a_ 10.5). Therefore, modification of lysines near the binding interface is avoided at labeling efficiencies below one fluorophore per B30.2 domain. Proteins were separated from free dye by size exclusion chromatography on a Superdex 200 10/300 column. Coupling efficiencies were calculated from the absorbances at 280 nm and 650/488 nm, respectively. The average number of dyes per molecule ranged from 0.3 to 0.45. Tryptic digestion of labeled rhTRIM5α B30.2 and matrix-assisted laser desorption ionization mass spectrometry (MALDI-MS) analysis of fluorescent cleavage products after reversed-phase chromatography revealed a single peak corresponding to the N-terminal B30.2 fragment GPSDMFR coupled to Alexa 488, confirming the N terminus as the major site of labeling.

### MST.

Twenty-five nanomolar concentrations of labeled B30.2 homologs were titrated with a 2:1 dilution series of the different capsid proteins. The samples were loaded into standard capillaries (NanoTemper Technologies GmbH), sealed with soft wax, and equilibrated by incubation at 7°C for 4 h before the measurement. Microscale thermophoresis (MST) measurements were carried out on a NanoTemper Monolith NT.115 instrument (NanoTemper Technologies GmbH) using 40% LED and 20% laser power with the laser switched on for 30 s, followed by an off period of 5 s. Fifteen or 16 thermophoresis measurements were carried out from three independently prepared dilution series. Samples from each dilution series were recorded twice. The raw data were analyzed using the NanoTemper software to obtain binding curves. *K_d_*s were calculated with Prism (GraphPad Software) by fitting the data with a 1:1 binding isotherm equation:

(1) $$f_{b}=[\text{capsid}]/\left(K_{d}+\left[\text{capsid}\right]\right)$$

where *f_b_* is the fraction of bound B30.2 domain, [capsid] is the capsid concentration, and *K_d_* is the dissociation constant. The MST experiments were carried out at 7°C, because higher capsid concentrations could be achieved without aggregation. To rule out effects of temperature on the binding affinity, measurements at 7°C and room temperature were compared, and very similar affinities were obtained (Fig. 5a).

### 2fFCS.

For dual-focus fluorescence correlation spectroscopy (2fFCS) measurements (44), a capsid dilution series such as that described for MST was prepared with Alexa 488-labeled TRIM5α B30.2 and CypA at a final concentration of labeled protein of 2 nM. CypA was included as a reference to quantify the contribution of viscosity to the measured diffusivity. CypA is suitable as a reference for several reasons. First, it has a similar molecular mass (19 kDa for Flag-CypA) and shape as rhTRIM5α B30.2 (23 kDa) and therefore a similar hydrodynamic radius (*R_h_*). Second, CypA and the B30.2 domain occupy overlapping binding surfaces on the capsid (26, 29–33), so their respective capsid complexes should also have similar *R_h_* values. Third, CypA has a relatively high affinity for the HIV-1 capsid surrogates (see Table 1), so binding occurs in a concentration range that is well separated from viscosity effects, which start to interfere with the measurements at capsid concentrations above ∼100 μM (Fig. 7). After completion of the 2fFCS measurement, the capsid concentration in each sample was determined via absorbance at 280 nm.

The measurements were carried out at 7°C on a confocal single-molecule instrument (MT200; PicoQuant), equipped with a differential interference contrast prism (U-DICTHC; Olympus). The sample was excited alternatingly with two orthogonally polarized diode lasers at 485 nm (LDH-D-C-485; PicoQuant) with a repetition rate of 40 MHz and a laser power of 30 μW each. The distance, *d*, of 436 ± 10 nm between the two foci in aqueous solution was determined by measuring a series of reference samples with a known diffusion coefficient (58). A custom-built temperature-controlled sample holder was used to cool the sample cell. The microscope objective was cooled as well to minimize the temperature gradient between sample cell and objective.

The fluorescence intensity cross- and autocorrelation curves obtained from the data of each focus were analyzed using a custom-written C++ routine and Mathematica (Wolfram Research) to obtain diffusion coefficients, *D* (59). These were converted to hydrodynamic radii, *R_h_*, with the Stokes-Einstein relation *R_h_* = *k_B_T*/(6πη*D*), where *k_B_* is the Boltzmann constant, *T* is the temperature, and η is the viscosity of the solution. The increase in apparent *R_h_* of the labeled molecules upon binding to capsid was analyzed with a simple stoichiometric binding model multiplied with a correction term for the increased viscosity at high capsid concentrations.

(2) $$R_{h}=(R_{h,0}+\frac{(R_{h,end}-R_{h,0})\cdot \left[\text{capsid}\right]}{K_{d}+\left[\text{capsid}\right]})\cdot \frac{\eta_{\left[\text{capsid}\right]}}{\eta_{0}}$$

In a first step, the CypA data set was fitted to extract the hydrodynamic radius of the unbound molecule (*R_h_*_,0_), the hydrodynamic radius of the capsid complex (*R_h_*_,end_), the dissociation constant (*K_d_*), and the capsid concentration-dependent viscosity contribution η_[capsid]_ empirically approximated by η_[capsid]_ = η_0_ (1 + [capsid]^β^, with η_0_ being the buffer viscosity, β adjusted in the range of 1.5 to 2 to fit the data, and [capsid] in μM). The binding of capsid to CypA is largely saturated before the sample viscosity starts to increase significantly, so the contributions of binding and viscosity to the increase in *R_h_* can be separated (Fig. 7a to c). For fitting the TRIM5α B30.2 2fFCS data, the parameters *R_h_*_,0_, *R_h_*_,end_, and η_[capsid]_ were fixed to the values extracted from fitting the CypA data set, so *K_d_* was the only free fit parameter. For comparison with the MST data, *R_h_* was converted to the fraction of bound B30.2 domain/CypA, *f_b_*, according to the equation:

(3) $$f_{b}=(\frac{R_{h}\eta_{0}}{\eta_{[\mathrm{capsid}]}}-R_{h,0})/\left(R_{h,\text{end}}-R_{h,0}\right)$$

## References

[1] Uchil PD, Quinlan BD, Chan WT, Luna JM, Mothes W 2008 TRIM E3 ligases interfere with early and late stages of the retroviral life cycle. PLoS Pathog 4:e16. doi:10.1371/journal.ppat.0040016. 18248090 PMC2222954

[2] Stremlau M, Owens CM, Perron MJ, Kiessling M, Autissier P, Sodroski J 2004 The cytoplasmic body component TRIM5alpha restricts HIV-1 infection in Old World monkeys. Nature 427:848–853. doi:10.1038/nature02343. 14985764

[3] Sayah DM, Sokolskaja E, Berthoux L, Luban J 2004 Cyclophilin A retrotransposition into TRIM5 explains owl monkey resistance to HIV-1. Nature 430:569–573. doi:10.1038/nature02777. 15243629

[4] Grütter MG, Luban J 2012 TRIM5 structure, HIV-1 capsid recognition, and innate immune signaling. Curr Opin Virol 2:142–150. doi:10.1016/j.coviro.2012.02.003. 22482711 PMC3322363

[5] Diaz-Griffero F, Li X, Javanbakht H, Song B, Welikala S, Stremlau M, Sodroski J 2006 Rapid turnover and polyubiquitylation of the retroviral restriction factor TRIM5. Virology 349:300–315. doi:10.1016/j.virol.2005.12.040. 16472833

[6] Li X, Sodroski J 2008 The TRIM5alpha B-box 2 domain promotes cooperative binding to the retroviral capsid by mediating higher-order self-association. J Virol 82:11495–11502. doi:10.1128/JVI.01548-08. 18799578 PMC2583650

[7] Diaz-Griffero F, Qin XR, Hayashi F, Kigawa T, Finzi A, Sarnak Z, Lienlaf M, Yokoyama S, Sodroski J 2009 A B-box 2 surface patch important for TRIM5alpha self-association, capsid binding avidity, and retrovirus restriction. J Virol 83:10737–10751. doi:10.1128/JVI.01307-09. 19656869 PMC2753111

[8] Wagner JM, Roganowicz MD, Skorupka K, Alam SL, Christensen D, Doss G, Wan Y, Frank GA, Ganser-Pornillos BK, Sundquist WI, Pornillos O 2016 Mechanism of B-box 2 domain-mediated higher-order assembly of the retroviral restriction factor TRIM5alpha. eLife 5:e16309. 27253059 10.7554/eLife.16309 PMC4936894

[9] Langelier CR, Sandrin V, Eckert DM, Christensen DE, Chandrasekaran V, Alam SL, Aiken C, Olsen JC, Kar AK, Sodroski JG, Sundquist WI 2008 Biochemical characterization of a recombinant TRIM5alpha protein that restricts human immunodeficiency virus type 1 replication. J Virol 82:11682–11694. doi:10.1128/JVI.01562-08. 18799573 PMC2583683

[10] Pertel T, Hausmann S, Morger D, Züger S, Guerra J, Lascano J, Reinhard C, Santoni FA, Uchil PD, Chatel L, Bisiaux A, Albert ML, Strambio-De-Castillia C, Mothes W, Pizzato M, Grütter MG, Luban J 2011 TRIM5 is an innate immune sensor for the retrovirus capsid lattice. Nature 472:361–365. doi:10.1038/nature09976. 21512573 PMC3081621

[11] Zhao G, Ke D, Vu T, Ahn J, Shah VB, Yang R, Aiken C, Charlton LM, Gronenborn AM, Zhang P 2011 Rhesus TRIM5α disrupts the HIV-1 capsid at the inter-hexamer interfaces. PLoS Pathog 7:e1002009. doi:10.1371/journal.ppat.1002009. 21455494 PMC3063768

[12] Weinert C, Morger D, Djekic A, Grutter MG, Mittl PR 2015 Crystal structure of TRIM20 C-terminal coiled-coil/B30.2 fragment: implications for the recognition of higher order oligomers. Sci Rep 5:10819. doi:10.1038/srep10819. 26043233 PMC4455283

[13] Sanchez JG, Okreglicka K, Chandrasekaran V, Welker JM, Sundquist WI, Pornillos O 2014 The tripartite motif coiled-coil is an elongated antiparallel hairpin dimer. Proc Natl Acad Sci U S A 111:2494–2499. doi:10.1073/pnas.1318962111. 24550273 PMC3932864

[14] Goldstone DC, Walker PA, Calder LJ, Coombs PJ, Kirkpatrick J, Ball NJ, Hilditch L, Yap MW, Rosenthal PB, Stoye JP, Taylor IA 2014 Structural studies of postentry restriction factors reveal antiparallel dimers that enable avid binding to the HIV-1 capsid lattice. Proc Natl Acad Sci U S A 111:9609–9614. doi:10.1073/pnas.1402448111. 24979782 PMC4084454

[15] Sebastian S, Luban J 2005 TRIM5alpha selectively binds a restriction-sensitive retroviral capsid. Retrovirology 2:40. doi:10.1186/1742-4690-2-40. 15967037 PMC1166576

[16] Stremlau M, Perron M, Welikala S, Sodroski J 2005 Species-specific variation in the B30.2(SPRY) domain of TRIM5alpha determines the potency of human immunodeficiency virus restriction. J Virol 79:3139–3145. doi:10.1128/JVI.79.5.3139-3145.2005. 15709033 PMC548447

[17] Javanbakht H, Yuan W, Yeung DF, Song B, Diaz-Griffero F, Li Y, Li X, Stremlau M, Sodroski J 2006 Characterization of TRIM5alpha trimerization and its contribution to human immunodeficiency virus capsid binding. Virology 353:234–246. doi:10.1016/j.virol.2006.05.017. 16808955

[18] Ganser-Pornillos BK, Chandrasekaran V, Pornillos O, Sodroski JG, Sundquist WI, Yeager M 2011 Hexagonal assembly of a restricting TRIM5alpha protein. Proc Natl Acad Sci U S A 108:534–539. doi:10.1073/pnas.1013426108. 21187419 PMC3021009

[19] Li YL, Chandrasekaran V, Carter SD, Woodward CL, Christensen DE, Dryden KA, Pornillos O, Yeager M, Ganser-Pornillos BK, Jensen GJ, Sundquist WI 2016 Primate TRIM5 proteins form hexagonal nets on HIV-1 capsids. Elife 5:e16269. doi:10.7554/eLife.16269. 27253068 PMC4936896

[20] Hatziioannou T, Cowan S, Goff SP, Bieniasz PD, Towers GJ 2003 Restriction of multiple divergent retroviruses by Lv1 and Ref1. EMBO J 22:385–394. doi:10.1093/emboj/cdg042. 12554640 PMC140727

[21] Yap MW, Nisole S, Lynch C, Stoye JP 2004 Trim5alpha protein restricts both HIV-1 and murine leukemia virus. Proc Natl Acad Sci U S A 101:10786–10791. doi:10.1073/pnas.0402876101. 15249690 PMC490012

[22] Perron MJ, Stremlau M, Song B, Ulm W, Mulligan RC, Sodroski J 2004 TRIM5alpha mediates the postentry block to N-tropic murine leukemia viruses in human cells. Proc Natl Acad Sci U S A 101:11827–11832. doi:10.1073/pnas.0403364101. 15280539 PMC511059

[23] Sastri J, Campbell EM 2011 Recent insights into the mechanism and consequences of TRIM5alpha retroviral restriction. AIDS Res Hum Retroviruses 27:231–238. doi:10.1089/aid.2010.0367. 21247355 PMC3048830

[24] Keckesova Z, Ylinen LM, Towers GJ 2004 The human and African green monkey TRIM5alpha genes encode Ref1 and Lv1 retroviral restriction factor activities. Proc Natl Acad Sci U S A 101:10780–10785. doi:10.1073/pnas.0402474101. 15249687 PMC490011

[25] Wilson SJ, Webb BL, Maplanka C, Newman RM, Verschoor EJ, Heeney JL, Towers GJ 2008 Rhesus macaque TRIM5 alleles have divergent antiretroviral specificities. J Virol 82:7243–7247. doi:10.1128/JVI.00307-08. 18480454 PMC2446970

[26] Fletcher AJ, Towers GJ 2013 Inhibition of retroviral replication by members of the TRIM protein family. Curr Top Microbiol Immunol 371:29–66. doi:10.1007/978-3-642-37765-5_2. 23686231

[27] Stremlau M, Perron M, Lee M, Li Y, Song B, Javanbakht H, Diaz-Griffero F, Anderson DJ, Sundquist WI, Sodroski J 2006 Specific recognition and accelerated uncoating of retroviral capsids by the TRIM5alpha restriction factor. Proc Natl Acad Sci U S A 103:5514–5519. doi:10.1073/pnas.0509996103. 16540544 PMC1459386

[28] Yap MW, Nisole S, Stoye JP 2005 A single amino acid change in the SPRY domain of human Trim5alpha leads to HIV-1 restriction. Curr Biol 15:73–78. doi:10.1016/j.cub.2004.12.042. 15649369

[29] Biris N, Tomashevski A, Bhattacharya A, Diaz-Griffero F, Ivanov DN 2013 Rhesus monkey TRIM5alpha SPRY domain recognizes multiple epitopes that span several capsid monomers on the surface of the HIV-1 mature viral core. J Mol Biol 425:5032–5044. doi:10.1016/j.jmb.2013.07.025. 23886867 PMC4116666

[30] Owens CM, Song B, Perron MJ, Yang PC, Stremlau M, Sodroski J 2004 Binding and susceptibility to postentry restriction factors in monkey cells are specified by distinct regions of the human immunodeficiency virus type 1 capsid. J Virol 78:5423–5437. doi:10.1128/JVI.78.10.5423-5437.2004. 15113921 PMC400345

[31] Maillard PV, Zoete V, Michielin O, Trono D 2011 Homology-based identification of capsid determinants that protect HIV1 from human TRIM5alpha restriction. J Biol Chem 286:8128–8140. doi:10.1074/jbc.M110.187609. 21169362 PMC3048699

[32] Nomaguchi M, Yokoyama M, Kono K, Nakayama EE, Shioda T, Doi N, Fujiwara S, Saito A, Akari H, Miyakawa K, Ryo A, Ode H, Iwatani Y, Miura T, Igarashi T, Sato H, Adachi A 2013 Generation of rhesus macaque-tropic HIV-1 clones that are resistant to major anti-HIV-1 restriction factors. J Virol 87:11447–11461. doi:10.1128/JVI.01549-13. 23966385 PMC3807366

[33] Gamble TR, Vajdos FF, Yoo S, Worthylake DK, Houseweart M, Sundquist WI, Hill CP 1996 Crystal structure of human cyclophilin A bound to the amino-terminal domain of HIV-1 capsid. Cell 87:1285–1294. doi:10.1016/S0092-8674(00)81823-1. 8980234

[34] Biris N, Yang Y, Taylor AB, Tomashevski A, Guo M, Hart PJ, Diaz-Griffero F, Ivanov DN 2012 Structure of the rhesus monkey TRIM5alpha PRYSPRY domain, the HIV capsid recognition module. Proc Natl Acad Sci U S A 109:13278–13283. doi:10.1073/pnas.1203536109. 22847415 PMC3421187

[35] Zhao G, Perilla JR, Yufenyuy EL, Meng X, Chen B, Ning J, Ahn J, Gronenborn AM, Schulten K, Aiken C, Zhang P 2013 Mature HIV-1 capsid structure by cryo-electron microscopy and all-atom molecular dynamics. Nature 497:643–646. doi:10.1038/nature12162. 23719463 PMC3729984

[36] Yang H, Ji X, Zhao G, Ning J, Zhao Q, Aiken C, Gronenborn AM, Zhang P, Xiong Y 2012 Structural insight into HIV-1 capsid recognition by rhesus TRIM5alpha. Proc Natl Acad Sci U S A 109:18372–18377. doi:10.1073/pnas.1210903109. 23091002 PMC3494900

[37] Gamble TR, Yoo S, Vajdos FF, von Schwedler UK, Worthylake DK, Wang H, McCutcheon JP, Sundquist WI, Hill CP 1997 Structure of the carboxyl-terminal dimerization domain of the HIV-1 capsid protein. Science 278:849–853. doi:10.1126/science.278.5339.849. 9346481

[38] Pornillos O, Ganser-Pornillos BK, Kelly BN, Hua Y, Whitby FG, Stout CD, Sundquist WI, Hill CP, Yeager M 2009 X-ray structures of the hexameric building block of the HIV capsid. Cell 137:1282–1292. doi:10.1016/j.cell.2009.04.063. 19523676 PMC2840706

[39] Pornillos O, Ganser-Pornillos BK, Yeager M 2011 Atomic-level modelling of the HIV capsid. Nature 469:424–427. doi:10.1038/nature09640. 21248851 PMC3075868

[40] Ganser-Pornillos BK, Cheng A, Yeager M 2007 Structure of full-length HIV-1 CA: a model for the mature capsid lattice. Cell 131:70–79. doi:10.1016/j.cell.2007.08.018. 17923088

[41] del Alamo M, Rivas G, Mateu MG 2005 Effect of macromolecular crowding agents on human immunodeficiency virus type 1 capsid protein assembly in vitro. J Virol 79:14271–14281. doi:10.1128/JVI.79.22.14271-14281.2005. 16254362 PMC1280224

[42] Yoshimura FK, Diem K, Learn GH Jr, Riddell S, Corey L 1996 Intrapatient sequence variation of the gag gene of human immunodeficiency virus type 1 plasma virions. J Virol 70:8879–8887. 8971017 10.1128/jvi.70.12.8879-8887.1996 PMC190985

[43] Jerabek-Willemsen M, Andre T, Wanner R, Roth HM, Duhr S, Baaske P, Breitsprecher D 2014 Microscale thermophoresis: interaction analysis and beyond. J Mol Struct 1077:101–113. doi:10.1016/j.molstruc.2014.03.009.

[44] Dertinger T, Pacheco V, von der Hocht I, Hartmann R, Gregor I, Enderlein J 2007 Two-focus fluorescence correlation spectroscopy: a new tool for accurate and absolute diffusion measurements. Chemphyschem 8:433–443. doi:10.1002/cphc.200600638. 17269116

[45] Yoo S, Myszka DG, Yeh C, McMurray M, Hill CP, Sundquist WI 1997 Molecular recognition in the HIV-1 capsid/cyclophilin A complex. J Mol Biol 269:780–795. doi:10.1006/jmbi.1997.1051. 9223641

[46] Li X, Yeung DF, Fiegen AM, Sodroski J 2011 Determinants of the higher order association of the restriction factor TRIM5alpha and other tripartite motif (TRIM) proteins. J Biol Chem 286:27959–27970. doi:10.1074/jbc.M111.260406. 21680743 PMC3151041

[47] Sawyer SL, Wu LI, Emerman M, Malik HS 2005 Positive selection of primate TRIM5alpha identifies a critical species-specific retroviral restriction domain. Proc Natl Acad Sci U S A 102:2832–2837. doi:10.1073/pnas.0409853102. 15689398 PMC549489

[48] Song B, Javanbakht H, Perron M, Park DH, Stremlau M, Sodroski J 2005 Retrovirus restriction by TRIM5alpha variants from Old World and New World primates. J Virol 79:3930–3937. doi:10.1128/JVI.79.7.3930-3937.2005. 15767395 PMC1061569

[49] Song B, Gold B, O'Huigin C, Javanbakht H, Li X, Stremlau M, Winkler C, Dean M, Sodroski J 2005 The B30.2(SPRY) domain of the retroviral restriction factor TRIM5alpha exhibits lineage-specific length and sequence variation in primates. J Virol 79:6111–6121. doi:10.1128/JVI.79.10.6111-6121.2005. 15857996 PMC1091705

[50] Kovalskyy DB, Ivanov DN 2014 Recognition of the HIV capsid by the TRIM5alpha restriction factor is mediated by a subset of pre-existing conformations of the TRIM5alpha SPRY domain. Biochemistry 53:1466–1476. doi:10.1021/bi4014962. 24506064 PMC4119003

[51] Salmon P, Trono D 2007 Production and titration of lentiviral vectors. Current Protoc Hum Genet Chapter 12:Unit 12.10. doi:10.1002/0471142905.hg1210s54. 18428406

[52] Gross I, Hohenberg H, Krausslich HG 1997 In vitro assembly properties of purified bacterially expressed capsid proteins of human immunodeficiency virus. Eur J Biochem 249:592–600. doi:10.1111/j.1432-1033.1997.t01-1-00592.x. 9370371

[53] Byeon I-JL, Meng X, Jung J, Zhao G, Yang R, Ahn J, Shi J, Concel J, Aiken C, Zhang P, Gronenborn AM 2009 Structural convergence between Cryo-EM and NMR reveals intersubunit interactions critical for HIV-1 capsid function. Cell 139:780–790. doi:10.1016/j.cell.2009.10.010. 19914170 PMC2782912

[54] Schuck P 2000 Size-distribution analysis of macromolecules by sedimentation velocity ultracentrifugation and lamm equation modeling. Biophys J 78:1606–1619. doi:10.1016/S0006-3495(00)76713-0. 10692345 PMC1300758

[55] Geertsma ER, Dutzler R 2011 A versatile and efficient high-throughput cloning tool for structural biology. Biochemistry 50:3272–3278. doi:10.1021/bi200178z. 21410291

[56] Berger I, Fitzgerald DJ, Richmond TJ 2004 Baculovirus expression system for heterologous multiprotein complexes. Nat Biotechnol 22:1583–1587. doi:10.1038/nbt1036. 15568020

[57] Weinert C, Grutter C, Roschitzki-Voser H, Mittl PR, Grutter MG 2009 The crystal structure of human pyrin b30.2 domain: implications for mutations associated with familial Mediterranean fever. J Mol Biol 394:226–236. doi:10.1016/j.jmb.2009.08.059. 19729025

[58] Hofmann H, Soranno A, Borgia A, Gast K, Nettels D, Schuler B 2012 Polymer scaling laws of unfolded and intrinsically disordered proteins quantified with single-molecule spectroscopy. Proc Natl Acad Sci U S A 109:16155–16160. doi:10.1073/pnas.1207719109. 22984159 PMC3479594

[59] Benke S, Roderer D, Wunderlich B, Nettels D, Glockshuber R, Schuler B 2015 The assembly dynamics of the cytolytic pore toxin ClyA. Nat Commun 6:6198. doi:10.1038/ncomms7198. 25652783 PMC4347018

